# Prevalence and risk factors for sexual assault among class 6 female students in unplanned settlements of Nairobi, Kenya: Baseline analysis from the IMPower & Sources of Strength cluster randomized controlled trial

**DOI:** 10.1371/journal.pone.0213359

**Published:** 2019-06-06

**Authors:** Michael Baiocchi, Rina Friedberg, Evan Rosenman, Mary Amuyunzu-Nyamongo, Gabriel Oguda, Dorothy Otieno, Clea Sarnquist

**Affiliations:** 1 Stanford Prevention Research Center, Palo Alto, California, United States of America; 2 Stanford University, Department of Statistics, Stanford, California, United States of America; 3 Africa Institute for Health and Development, Nairobi, Kenya; 4 Stanford University School of Medicine, Department of Pediatrics, Stanford, California, United States of America; University of Texas Medical Branch at Galveston, UNITED STATES

## Abstract

**Background:**

Gender-based violence (GBV) is a crucial global health problem among all age groups, including adolescents. This study describes incidences of GBV, as well as factors associated with sexual assault, among female adolescents in class six living in urban informal settlements in Nairobi, Kenya.

**Methods:**

Study participants were interviewed using a structured survey instrument focusing on experiences of GBV, including emotional, physical, and sexual violence, and corresponding perpetrators, as well as gender attitudes, alcohol use, self-efficacy, and previous sexual experiences. Summary statistics and clustered bootstrap confidence intervals were calculated for social behaviors and violence rates. Stepwise logistic regression identified variables associated with an adolescent’s experience of sexual assault.

**Findings:**

In this population 7·2% of adolescent girls reported being raped in the prior twelve months, with 11·1% of these rape victims reporting over five experiences. Among the 21·3% who report having had a boyfriend, 38·1% reported emotional, physical, and/or sexual intimate partner violence (IPV). Boyfriends were identified most often as perpetrators, accounting for 46·3% of reported lifetime rapes. Previous experience of physical (p = <0·001) or emotional (p<0·001) IPV and home violence (p<0·001) were risk factors for being raped, while high self-efficacy (p<0·001) was a protective factor.

**Interpretation:**

Sexual assault and GBV are major challenges in this highly-disadvantaged population. Novel prevention efforts are needed for this age group, as prevention is often targeted at older adolescents. Prevention efforts should focus on assaults by perpetrators known to adolescents, especially boyfriends, and may need to account for the adolescents’ previous experience of, and exposure to, violence.

## Background

Evidence suggests that rates of gender-based violence (GBV) and sexual assault against adolescents are high in many settings [1, 2]. Such adverse and traumatic experiences result in many negative outcomes for survivors, including serious and permanent physical and psychological damage such as sexually transmitted illnesses, infertility, depression, and post-traumatic stress disorder [3–5]. Thus, it is essential to understand the incidence of, and risk factors for, violence, in order to develop and evaluate prevention approaches for this population [6].

Prevention of gender based violence among adolescents is increasingly an interest of practitioners and researchers, although the vast majority of existing studies are focused on adults, are on intimate partner violence, and/or focus on high income settings [7]. Nonetheless, there is some previous data. The most relevant study to the present work is one on students in grade 8 (aged 12–19 years) around Pretoria, South Africa [8], who reported a previous 12 month sexual intimate partner violence (IPV) rate of 13·8%. Another study in South Africa, in an older population (18–34 years of age) in informal settlements around Durban, reported 9·8% incidence of sexual IPV in the previous 3 months [9]. A study in Cote d’Ivoire reported sexual IPV rates of 10·5–13·8% across two different study arms, both in women over 18 years of age [10]. A large, cross-sectional, multi-country study suggests that current IPV rates among adults vary widely, from 16–21% in a province in Tanzania to 42–46% countrywide in Ethiopia [11]. However, there is a paucity of evidence on the epidemiology of sexual violence committed by non-partners, as well as among younger adolescent populations [7].

Over half of the population of Nairobi, Kenya live in more than 200 urban informal settlements, or slums, which are among the largest in the world. These settlements are characterized by high population density, inadequate access to water and sanitation, and insecurity, including high rates of all kinds of violence. These settlements are continuing to grow quickly, as are other settlements in many regions and countries, including India and Brazil [12]. This rapid urbanization combined with high population growth in many parts of the world means that settlement-dwelling adolescents will be a key population to reach for violence prevention, as well as other health and wellbeing interventions, for the foreseeable future.

In Kenya, previous data has shown that the annual incidence of sexual assault among an open cohort of 5686 upper primary school female students aged 10–16 years in several informal settlements was 7·3% at baseline, and that perpetrators of multiple episodes of sexual violence were most likely to be boyfriends or relatives [13]. This study was a cluster-randomized trial of a similar intervention run in 2013–2014 in the same settlements as the current study. In contrast to the current study, Baiocchi et al. 2016 used (i) an open-cohort design, (ii) completely anonymous surveys (thus no linkage between baseline and endline surveys), (iii) had a restricted survey, and (iv) used the trainers to deliver the surveys (and thus was more susceptible to demand effects). No participants in the current study were in the Baiocchi et al. 2016 study. The current study looks at baseline incidence of sexual violence in a similar population, but distinguishes between IPV and non-partner violence, and, perhaps most importantly from a prevention perspective, quantifies associations between risk factors and experiences of sexual violence.

## Methods

### Study design and population

This data was collected as a baseline for a previously-described cluster-randomized trial (CRT) evaluating the impact of an empowerment self-defense based sexual assault prevention program with students in grade 6 [14]. Data for this analysis was collected from January 2016 through October 2016 in six informal settlements of Nairobi, Kenya: Dandora, Huruma, Kibera, Korogocho, Kariobangi, and Mukuru. Partners include No Means No Worldwide (NMNW), a US-based NGO that developed the intervention; Ujamaa Africa, a Nairobi-based NGO that implements the program in Nairobi; and the African Institute for Health and Development (AIHD), a research firm in Nairobi. The trial is registered on ClinicalTrials.gov: #NCT02771132.

As part of the larger CRT, 108 schools across the six settlements were matched on pre-trial information; after dropout, 95 schools were surveyed at baseline. One school in each pair was randomly assigned to the treatment group and the other was assigned standard of care (SOC). All students in the schools received the assigned training. However, within schools, students in class 6 were randomly sampled to participate in the surveys by drawing different colored beads from an opaque bag. Potential participants who drew the “participant beads”, up to 101 per school, were invited to participate, beginning with an informed assent and parental opt-out consent process.

### Ethics and safety

Surveys were administered and recorded by the Nairobi-based African Institute for Health and Development (AIHD). A panel of Stanford-based specialists with expertise in pediatrics, adolescent medicine, pediatric psychology and psychiatry, and global health, met regularly to provide oversight of the management and evaluation of the project. Data collection teams were trained at a two-day, all-day session prior to each period of data collection. The trainings, co-taught by Stanford University and AIHD, included ethical data collection procedures, trauma-informed interviewing techniques, cultural considerations and interpretations of surveys. The sessions were attended by all the AIHD data collectors, researchers from Stanford University, and members of Ujamaa-Africa (the NGO deploying the trainings).

In addition to the annual trainings, the AIHD data collection team had a settlement-specific manager in each community who supervised and mentored the data collection teams. Furthermore, if study participants decided to disclose to a trainer or a data collector that violence occurred, a Ujamaa-Africa trainer assessed the needs of the student and provided a referral to the location-appropriate medical provider (e.g., Médecins Sans Frontières) and/or legal advice provider.

### Measures

This study drew on several existing scales and survey instruments previously-used in this, or similar, populations and age groups. As validated scales for this age group and literacy level were not available for the primary outcomes and several secondary outcomes, the only previously-validated scale used was the “Self-Efficacy Questionnaire for Children” [15]. Self-efficacy in this age group encompasses three domains: social, emotional, and academic self-efficacy. For other outcomes, the research team adapted previously existing survey tools, including the Kenya Violence Against Children Survey and the Stepping Stones surveys, as described in more detail elsewhere [16].

### Data analysis

Several distinct data analyses were performed, including of: (i) demographics, (ii) rates of intimate partnerships and intimate partner violence (sexual, physical, and emotional), (iii) rates of sexual violence (IPV and non-IPV), (iv) gender norms, (v) perpetrators, and (vi) factors associated with reporting sexual assault.

Data about sexual assault was self-reported, which resulted in missing or conflicting reports in some cases. To address this, we aggregated information from many different questions about sexual assault in the survey and validated this method with an Adjudication Model. In addition, we used the clustered bootstrap method as a more accurate alternative to standard confidence intervals, which fail to account for the structure of this study’s sampling procedure [17].

Rape was defined as being forced to have sex against ones will, whether that force was physical, threats or intimidation, or coercion. Sexual assault was defined as any unwanted sexual act, including but not limited to rape, and including both IPV and non-partner violence. Physical IPV was defined as any time a boyfriend slapped, threw something at, shoved, hit, kicked, dragged, beat, choked, burned, threatened to use a weapon or used a weapon on a victim. Emotional IPV was defined as any time a boyfriend insulted, made fun of, humiliated, intentionally scared or threatened a victim, or otherwise made a victim feel bad about herself.

We conducted multivariable modelling to identify risk factors associated with being sexual assaulted using a multiple logistic regression model on centered and scaled data. Predictors were selected via forward and backward stepwise regression, evaluated by the Akaike Information Criterion (AIC) [18]. All variables with fewer than 5% of students in a category (such as frequent drug use, with <1% of students) were excluded from the logistic regression. We ran a principal components analysis on the self-efficacy questionnaire and found that the questions were similarly weighted; therefore we included all individual questions rather than only some questions or a summary score. Prior sexual experience was also not included; the survey asked about previous intercourse before rape in order to build trust with the adolescents, but did not delineate carefully between consensual sex and rape until asking about forced sexual activity. Therefore, previous intercourse was likely to be spuriously correlated with the outcome of interest, and was not included in the regression.

Confidence intervals are from a clustered bootstrap over 1000 replicates. Two-sided p-values are computed by a clustered permutation test, analogous to the clustered bootstrap except that we permuted the responses instead of re-sampling within clusters. Note that these confidence intervals and p-values test slightly different hypotheses. The bootstrap is based on person-to-person variability (e.g., Neyman-Pearson null hypotheses [19]), while the permutation test evaluates within-observation variability across the possible responses (e.g., Fisher’s sharp null hypothesis [20]).

### Role of the funding source

This research was supported by the South African Medical Research Council through the What Works to Prevent Violence Innovation Grant (#52069) as a result of the support obtained from the Secretary of State for International Development at the Department for International Development. Its contents are solely the responsibility of the authors and do not necessarily represent the official view of the Secretary of State for International Development at the Department for International Development or the South African Medical Research Council. This research was conducted with U.S. government support under and awarded by the Department of Defense, Air Force Office of Scientific Research, National Defense Science and Engineering Graduate (NDSEG) Fellowship, 32 CFR 168a. We are also grateful for support from the Marjorie Lozoff Fund, Michelle R. Clayman Institute for Gender Research, Stanford University.

## Results

### Demographics

A total of 4125 female participants from 94 schools completed the baseline survey (Table 1). The mean age was 11·7 years, with a range of 10–14 years (data not shown). About half of study participants lived in households of 3–5 people, with another third in 6–8 person households. Participants reported 16·5% (n = 4080) had either one or both parents deceased or missing. To measure socioeconomic status, the survey asked how easy it would be for an adolescent to get 1000 shillings (approximately 10 USD) for a family member to get medical attention or a hospital visit. Half of the girls rated it difficult or very difficult to get this money, while a quarter reported that they have medical insurance. Reported “regular use” of alcohol and other drugs were low, at 11·2% and 0·9%, respectively, and another 10·2% reporting infrequent alcohol use (data not shown).

**Table 1:** Baseline demographics.

	Count (n = 4125)	Percent
Household size	n = 4089	
0–2	151	3·7%
3–5	2308	56·4%
6–8	1343	32·8%
9–12	233	5·7%
13+	54	1·3%
Mother’s education level	n = 4110	
None	40	1.0%
Primary incomplete	230	5·6%
Primary school	364	8·9%
High school incomplete	249	6·1%
High school	724	17·6%
Post-high school	1185	28·8%
Do not know	1318	32·1%
Parental status	n = 4080	
Both alive	3408	83·5%
Mother deceased	104	2·5%
Father deceased	315	7·7%
Orphaned	60	1·5%
Do not know	193	4·7%
Ability to get 1000 shillings for hospital or medicine (n = 4111)		
Very difficult	774	18·8%
Difficult	1249	30·3%
Easy	848	20·6%
Very easy	245	6.0%
Have medical insurance	995	24·2%

### Experiences of violence

**Fig 1:**
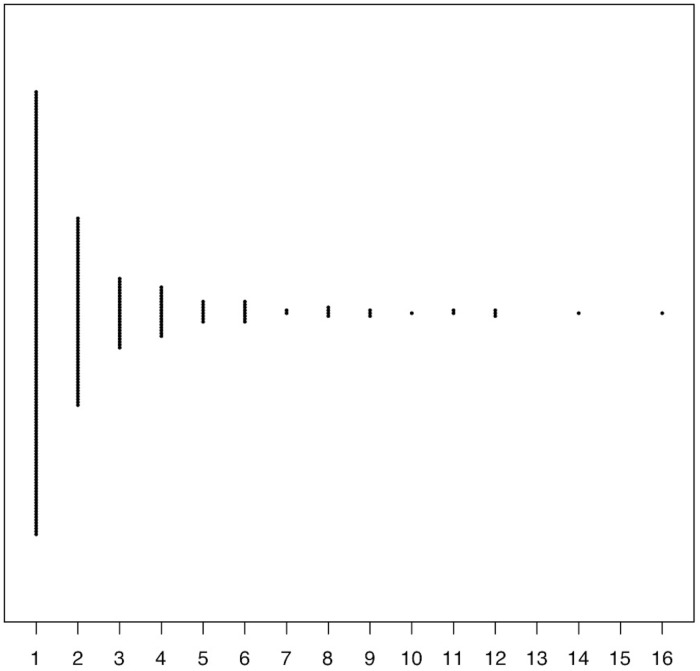
Bee swarm plot showing reported total counts of rape in the last 12 months, among individuals who were raped at least once in the last 12 months.

In this population, 11·0% (n = 4125) of girls reported sexual assault in the last twelve months, with 7·2% (n = 4125) reporting rape in the last twelve months (Fig 1). Of the 453 girls reporting sexual assault in the last twelve months, 57·2% (n = 402 due to missing answers) reported at least one attempted rape, with the remaining answers reporting completed rape or another form of sexual assault. The number of reported rapes in the last twelve months ranged from 1–16, with 52·3% (n = 298) of the girls reporting one rape and 10·4% (n = 298) reporting over five rapes. The number of reported lifetime rapes ranged from 1–60, with 51·7% (n = 412) of the girls reporting one lifetime rape, and 15·8% (n = 412) reporting over five rapes.

858 (21·3%) girls have had a boyfriend at some point in their lives (Table 2). Of those, in the last year, 33·3% reported experiencing emotional IPV, 21·7% ßreported physical IPV, and 16·7% reported sexual IPV. The denominator for IPV questions is the number of girls who have had a boyfriend, here 858. IPV reports are for IPV within the last 12 months. Of all respondents, 7·2% (n = 4125) reported rape in the last twelve months, while 9·9% (n = 4125) reported rape at some point in their lives. Incidence of witnessing violence at home was high, with 23·0% of girls seeing someone (father or other) hitting their mother.

**Table 2:** Baseline violence statistics.

	*Count (n = 4125)*	*Percent*	*95% Confidence Interval*
Have ever had a boyfriend	858 (n = 4094)	21·3%	(19·8%, 23·7%)
Violence at home	947 (n = 4125)	23·0%	(21·6%, 24·1%)
Sexual assault (12 months)	453 (n = 4125)	11·0%	(10·1%, 12·0%)
Rape (12 months)	298 (n = 4125)	7·2%	(6·4%, 8·1%)
Once	156 (n = 298)	52·3%	(46·9%, 57·4%)
2–4 times	111 (n = 298)	37·2%	(31·5%, 42·0%)
5+ times	31 (n = 298)	10·4%	(7·8%, 15·1%)
Sexual IPV	143 (n = 858)	16·7%	(14·9%, 19·1%)
Physical IPV	186 (n = 858)	21·7%	(18·6%, 23·4%)
Emotional IPV	268 (n = 858)	33·3%	(28·8%, 33·9%)

### Gender norms

Some negative gender norms were frequently accepted (Table 3), such as “a woman should listen to her husband” (54·3% strongly agree and 36·3% agree) and “if a woman wears miniskirts and drinks alcohol then she is asking for trouble” (44·8% strongly agree and 18·1% agree). Others were less accepted, such as “sometimes a man may have a good reason to hit his girlfriend” (14·8% strongly agree and 19·7% agree) and “if a wife does something wrong she should expect her husband to punish her” (10·0% strongly agree and 12·0% agree).

**Table 3:** Responses to questions concerning gender norms.

	Strongly Agree	Agree	Disagree	Strongly Disagree
If a woman drinks alcohol and wears miniskirts she is asking for trouble.	44.8% (1841)	18·1% (743)	11·9% (488)	25·2% (1036)
A woman should listen to her husband.	54·3% (2233)	36·3% (1493)	4·5% (187)	4·8% (197)
A woman has to teach her husband to respect her.	40·0% (1640)	30·2% (1239)	16·5% (676)	13·4% (551)
A woman should choose her own friends even if her boyfriend or husband disagrees.	28·8% (1178)	23·5% (962)	23·9% (979)	23·8% (974)
Men should share the work around the home such as doing the dishes or cleaning or cooking.	27·5% (1123)	22·8% (929)	22·0% (897)	27·8% (1133)
Sometimes a man may have a good reason to hit his girlfriend.	14·8% (603)	19·7% (803)	26·2% (1067)	39·4% (1607)
A woman can refuse to have sex with her husband if she does not want it for any reason.	34·7% (1416)	23·0% (937)	19·5% (797)	22·8% (928)
If a wife does something wrong she should expect her husband to punish her.	10·0% (409)	12·0% (489)	32·2% (1313)	45·8% (1870)
A woman has to know how to look after herself as she cannot rely on her man to care for her.	48·8% (1994)	26·6% (1088)	12·1% (495)	12·4% (506)
A man cannot control himself when he wants sex.	21·5% (879)	19·0% (777)	24·7% (1008)	34·8% (1422)
A woman should expect to be taught how to behave by her boyfriend.	16·8% (686)	17·0% (695)	26·4% (1081)	39·8% (1630)
A woman should not expect the fathers of her children to give her money.	22·9% (938)	20·8% (853)	24·0% (986)	32·4% (1328)

### Multivariable model: Risk and protective factors for rape in the last twelve months

Multivariable analysis identified several risk variables associated with reporting at least one rape in the last twelve months (Table 4). Particularly strong predictors included prior emotional IPV (coefficient = 0·23, 95% CI 0·11–0·34, p = 0·005), prior physical IPV (coefficient = 0·21, 95% CI 0·10–0·30, p = 0·005), violence at home (coefficient = 0·26, 95% CI 0·15–0·36, p = 0·001) and prior alcohol use (coefficient = 0·26, 95% CI 0·16–0·35, p = <0·001). Other selected risk factors included average agreement with the gender-normative opinions on statements in Table 3 (p = 0·06) and a mother being deceased (p = 0·2). The significant protective factors were a high emotional self-efficacy score (p<0·001), and the ability to get 1000 shillings for medical expenses was also protective (p = 0·05). Candidate predictors not selected by stepwise regression included a father being deceased and academic and social self-efficacy scores.

**Table 4:** Risk factors for experience of rape in the last twelve months, multivariable model.

	Coefficient	95% CI	p-value
Emotional IPV	0·23	(0·11, 0·34)	0·005
Physical IPV	0·21	(0·10, 0·30)	0·005
Prior alcohol use	0·26	(0·16, 0·35)	<0·001
Violence at home	0·26	(0·15, 0·36)	0·001
High emotional self-efficacy score	-0·24	(-0·35, -0·13)	<0·001
Mother deceased	0·12	(0·04, 0·19)	0·2
Average agreement with gender-normative statements	0·11	(0·00, 0·24)	0·06
Can get money to pay for hospital visit if needed	-0·12	(-0·26, 0.00)	0·05

### Rape perpetration

In the last twelve months, the survey asked only if a boyfriend or somebody else perpetrated a rape. To this question, 30·4% (n = 276) of respondents reported rape by a boyfriend, 25·0% by a man who was not a boyfriend, and 44·6% by both categories.

Of the girls (412) who reported a lifetime rape, 330 gave information about the perpetrator, and 306 of these reported how many times they had been raped by different perpetrators. The 330 individuals describing any perpetrator information reported 344 total perpetrators, of whom 65·7% (n = 344) were boyfriends (Table 5). Family members accounted for 13·1% of rapes, strangers 17·4%, and authority figures 3·8%. Counts of rapes by perpetrator from the 306 adolescents answering this question are given in Table 5. Percentages in Table 5 show how many of the individuals who reported assaults attributed at least one to a given category of perpetrator. Surveys reporting multiple perpetrators but without numbers of assaults by perpetrator are omitted from the percentage calculations, because there is not enough information to infer specific counts of perpetration. Surveys reporting multiple perpetrators that did specify the number of assaults by perpetrator are included.

**Table 5:** Lifetime perpetrator distribution.

All reports (n = 344)			Counts of rapes by perpetrator (n = 306)			
	Number of girls raped by this perpetrator	Percent	Mean	Median	Min	Max
Boyfriend	226	65·7%	2.6	2	1	30
Relative	45	13·1%	5.1	2	1	60
Authority	13	3·8%	1.9	1	1	5
Stranger	60	17·4%	2.2	1	1	11

## Discussion

This study reports prevalence and risk factors associated with rape and other forms of sexual assault among class 6 girls, 10–14 years old, in schools that serve informal settlements of Nairobi, Kenya. The annual rape incidence of 7·2% reported here is consistent with the previous studies of sexual assault in these populations, with rates ranging from (i) 7·3% in a study of the same age group in Nairobi settlements, to (ii) 11·4% in a large multi-country and multi-age study across Eastern Africa, to (iii) 13·8% in slightly older adolescents in South African settlements, to as high as (iv) 17·9% in older Kenyan adolescents in similar settlements [8, 13, 14, 21].

Identifying risk factors associated with experiencing sexual assault can help target prevention, as well as care and treatment, services. In the multivariable model, the most significant risk factors associated with experiencing sexual assault were related to having had previous experiences of violence (physical, emotional, or witnessed against mother). Some version of this finding has been reported in virtually every study considering these relationships; there is a detrimental cycle of those who have previously experienced violence being more likely to experience it again [22].

Self-efficacy, defined as “individual’s belief in his or her ability to engage in appropriate actions in a given scenario” [15], is broken into three categories with this scale: social, academic, and emotional. In this data, high emotional self-efficacy was highly protective against rape (p<0·001), while social and academic self-efficacy were not predictive. When aggregated to a single average, higher self-efficacy was again highly protective against rape (p<0·001, data not shown). This finding is consistent with the literature; higher self-efficacy has been shown in previous studies to co-occur with interventions that protect against sexual assault [23], and is also associated with being more likely to engage in a bystander intervention to potentially prevent someone else from being assaulted, both largely in North American university settings. The consistency of these prior results among adolescents indicates that interventions targeting self-efficacy may be useful across both younger and older adolescents. Furthermore, data suggests that previous sexual assault may diminish some components of self-efficacy [6]; this may be one way in which a previous assault experiences pre-disposes an individual to being the victim of future assaults.

Low average agreement with the gender-normative statements in Table 3 was selected as a protective factor against rape (p = 0·06), although not quite significant at the 0·05 level. One study in a similar age group found that only “negative styles of resolving partner conflict” was associated with higher risk of sexual IPV among girls, but “disagreements with dating abuse”, “disagreement with male sexual entitlement” and “negative styles of resolving general conflict” were not associated [24]. The broader preponderance of evidence, mostly among adult populations and largely at the community level, suggest that negative gender norms, especially related to accepting norms supporting violence against women, are associated with higher levels of both physical and sexual violence against women [25–27]. Furthermore, many sexual and physical violence prevention programs, especially those aimed at men, include a norms-changing component aimed at supporting pro-social behavior, and ultimately, social transformation [28, 29].

The largest category of perpetrators reported by the adolescents were boyfriends, who account for over half of the reported rapes. Nearly a quarter of this population reported having a boyfriend, which was higher than expected given the mean age of 11·7 years. As a result, rates of violence by intimate partners were high, with 21·2% and 31·0% reporting physical and emotional IPV, respectively. These numbers are similar to numbers from a study of older adolescents in South Africa, where 24·1% of respondents with a partner reported physical IPV and 40·5% reported emotional IPV [8]. Despite boyfriends being the most frequent perpetrator, the number of assaults by perpetrator was highest among relatives, with a mean of over 5 and a maximum of 60. It seems possible that relatives choose victims that cannot, or will not, report the assaults or be able to get away, resulting in serial opportunities for sexual assault.

These findings present several core insights for future research. First, perpetration by boyfriends was very high given the age group in the study; further understanding of the dynamic of these relationships should be explored. For example, if the perpetrators are primarily boys in the same age group, as compared to much older men, or if the majority of perpetrators are adolescents from different schools, this will help tailor future interventions. Second, empowerment interventions such as this one focus on a trainee’s self-efficacy. In these analyses, we cannot establish the direction of causality, but observing that high self-efficacy co-occurs with lower incidence of rape it is consistent with theoretical underpinnings of empowerment-based interventions. A detailed exploration of this causal pathway could also inform future intervention strategies.

### Diversity

This study focuses on adolescent girls living in five informal settlements outside of Nairobi. This population is very diverse among populations traditionally studied in major journals, especially because of their low socioeconomic status and because sexual violence studies often focus on older populations. The co-authors from Kenya worked carefully to ensure ethical contact with the study participants, informing the full team on cultural sensitivity and linguistic awareness. The study is targeted to this and similar populations, and while unlikely to be generalizable to more privileged populations, provides potential for generalization to other disadvantaged groups.

### Limitations

Several key limitations to this study include the cross-sectional nature of the data, the role of social desirability bias, and the low response rate to questions about perpetrator. First, as these are baseline and cross-sectional data, only associations can be established. However, in the follow-up to this study, which will include measurements at 12 months and 24 months after the intervention period, we should be able to establish causality. Second, social desirability bias may have played a large role in responses to many of these questions, including on sexual assault, all types of IPV, drug and alcohol use, and having a boyfriend. Attempts were made to limit social desirability bias by having confidential surveys and making it clear that data collectors (and peers) would not know answers, but may not have been sufficient in all cases. Third, for the question on perpetrators, about half of the adolescents who reported being assaulted refused to answer, and it is likely that those who didn’t answer were systematically different from those who did. In particular, it is possible that some types of perpetrators exert more pressure than other types of perpetrators to not report, leading to a different profile of perpetrators, in reality, than that reported here.

## Conclusions

This study fills significant gaps in the knowledge base about sexual assault and GBV among young female adolescents, in particular those living in urban informal settlements. The findings confirm both the high rates of violence experienced by this vulnerable adolescent population and the need for effective prevention strategies designed for them. The costs of these levels of violence, both in terms of health and well-being, but also monetary, to families, communities, and countries, is staggering. This data serves as a baseline for a large cluster randomized trial that will focus on the effects of an empowerment self-defense approach in this age group.

## Supporting information

S1 File This is the baseline survey provided to the girls. (PDF)
