# Supplementary material for: Prevalence and risk factors for sexual assault among class 6 female students in unplanned settlements of Nairobi, Kenya: Baseline analysis from the IMPower & Sources of Strength cluster randomized controlled trial
Source: PLoS One. 2019 Jun 6;14(6):e0213359. doi: 10.1371/journal.pone.0213359 (PMC6553848; doi:10.1371/journal.pone.0213359)
Supplement: S1 File — (PDF) [file pone.0213359.s001.pdf]

Girls Questionnaire

Youth ID: [ ][ ][ ][ ][ ]

School ID Number: [ ][ ][ ][ ][ ]

Interviewer Name:

Interview Date: __ __ / __ __ / __ __ __ __
Day Month Year

Interview StartTime: __ __ : __ __ : __ __

Interview EndTime: __ __ : __ __ : __ __

**Section 1: Background (Stepping Stones 1)**		
**No.**	**Questions and Filters**	**Coding Categories**
**101**	What is your current class at school?	○ Class 4<br>○ Class 5<br>○ Class 6<br>○ Class 7<br>○ Class 8
**102**	Have you ever taken a previous class that provided you with self-defense skills, such as boxing, martial arts, or tae kwon do	○ Yes ○ No ○ Don't Know
**103**	Have you taken No Means No Self-Defense classes before?	○ Yes ○ No ○ Don't Know
**104**	If yes, in what year was your most recent No Means No Self-Defense Class?	Year 2 0 _ _ ○ N/A
**105**	If yes, where was your most recent No Means No Self-Defense Class?<br><br>Write legibly and only in capital letters.<br><br>If "Not Applicable" please mark the bubble marked "N/A"	○ N/A
**106**	Has anyone taught you skills from a No Means No class in the past 12 months?	○ Yes ○ No ○ Don't Know
**107**	If yes, who taught you those skills?<br><br>If "Other" please identify them in the text boxes provided	○ Friend ○ Classmate ○ Teacher ○ N/A<br>○ Sister ○ Other ○ Don't Know

**108**	If a person became ill in your home and 1000 Shillings were needed for treatment or medicines, would you say it would be very easy, easy, quite difficult or very difficult to find the money?	○ Very Difficult ○ Difficult ○ Easy ○ Very Easy ○ Have Medical Insurance
**119**	How many people, including all children, live in your home?	☐☐ ○ N/A
**110**	How many brothers live with you?	☐☐ ○ N/A
**111**	How many sisters live with you?	☐☐ ○ N/A
**112**	Has your mother died?	○ Yes ○ No ○ Don't Know
**113**	Has your father died?	○ Yes ○ No ○ Don't Know
**114**	What is/was your mother's highest education level?	○ No Schooling ○ Primary School Incomplete ○ Primary School Complete ○ High School Incomplete ○ High School Complete ○ Post High School Qualification ○ Don't Know

Family Life (FL)

Please make an X in the bubble of the best answer

**FL1**	**In the last 2 weeks,** how many times did you go to school without breakfast because of lack of food at home?	○ Never ○ Sometimes ○ About once a week ○ All or most days
**FL2**	**In the last 2 weeks,** how many times did you go to sleep without dinner because of lack of food at home?	○ Never ○ Sometimes ○ About once a week ○ All or most days
**FL3**	Can/could your mother read **AND** write?	○ No ○ She reads only ○ She reads and writes ○ Don't Know
**FL4**	Can/could your father read **AND** write?	○ No ○ He reads only ○ He reads and writes ○ Don't Know
**FL5**	What is the floor of your house made of? If not mud or cement, please specify the material in the provided text box.	○ Mud ○ Cement ○ Don't Know<br>Other Material<br>☐☐☐☐☐☐☐☐☐☐☐☐☐☐☐☐☐☐☐☐☐
**FL6**	Do you have electricity for lights?	○ Yes ○ No
**FL7**	Do you have gas for cooking?	○ Yes ○ No

**FL8**	How far from your house do you have to go for drinking water?	○ Very Near ○ Near ○ Somewhat Far ○ Far ○ Very Far
**FL9**	Do you get water at home? How long does it take you to get water, in minutes?	○ Yes ○ No<br># Minutes: ☐☐
**FL10**	How many rooms do you have at home, including bathrooms?	☐☐
**FL11**	Do you have a working television at home?	○ Yes ○ No

Peer Victimization Scale (PVS)

Below is a list of things that some children do to other children. Please make an X in the bubble of the best answer.

How many times within the past 2 weeks has another child done these things to you?

**PVS1**	Called me bad names	○ Never	○ Once	○ 2-3 times	○ 4+ times
**PVS2**	Tried to get me in trouble with my friends	○ Never	○ Once	○ 2-3 times	○ 4+ times
**PVS3**	Took something of mine without permission	○ Never	○ Once	○ 2-3 times	○ 4+ times
**PVS4**	Made fun of me because of my appearance	○ Never	○ Once	○ 2-3 times	○ 4+ times
**PVS5**	Made fun of me for some reason other than my appearance	○ Never	○ Once	○ 2-3 times	○ 4+ times
**PVS6**	Tripped me to make me fall	○ Never	○ Once	○ 2-3 times	○ 4+ times
**PVS7**	Pushed me to hurt me	○ Never	○ Once	○ 2-3 times	○ 4+ times
**PVS8**	Hurt me physically	○ Never	○ Once	○ 2-3 times	○ 4+ times
**PVS9**	Beat me so badly that I was injured	○ Never	○ Once	○ 2-3 times	○ 4+ times
**PVS10**	Deliberately broken something that belongs to me	○ Never	○ Once	○ 2-3 times	○ 4+ times
**PVS11**	Tried to make other children turn against me	○ Never	○ Once	○ 2-3 times	○ 4+ times
**PVS12**	Stole something from me	○ Never	○ Once	○ 2-3 times	○ 4+ times
**PVS13**	Refused to talk to me	○ Never	○ Once	○ 2-3 times	○ 4+ times
**PVS14**	Made other people not talk to me	○ Never	○ Once	○ 2-3 times	○ 4+ times

**PVS15**	Deliberately damaged something of mine	○ Never	○ Once	○ 2-3 times	○ 4+ times

Corporal Punishment School (CPS)
Please make an X in the bubble of the best answer.
How many times within the past 2 weeks…

**CPS1**	Were you slapped, hit, beaten, or physically punished by a teacher?	○ Never	○ Once	○ 2-3 times	○ 4+ times
**CPS2**	Have you been beaten so hard at school that you were injured?	○ Never	○ Once	○ 2-3 times	○ 4+ times
**CPS3**	Did a teacher hit you with a stick, whip, strap, or other hard object?	○ Never	○ Once	○ 2-3 times	○ 4+ times

Physical Punishment At Home (PPH)
Please make an X in the bubble of the best answer.
How many times within the past 2 weeks…

**PPH1**	Were you slapped, hit, beaten, or otherwise physically punished by a parent/guardian?	○ Never	○ Once	○ 2-3 times	○ 4+ times
**PPH2**	Have you been beaten so hard at home that you were injured?	○ Never	○ Once	○ 2-3 times	○ 4+ times
**PPH3**	Did a parent/guardian hit you with a stick, whip, strap, or other hard object?	○ Never	○ Once	○ 2-3 times	○ 4+ times

Parent/Guardian Fighting (PF) & Abuse of Mother
Please make an X in the bubble of the best answer.
How many times within the past 2 weeks…

**PF1**	Have you seen or heard that your father/male guardian had a physical fight with another man?	○ Never	○ Once	○ 2-3 times	○ 4+ times
**PF2**	Have you seen or heard your father/male guardian hit your mother/female guardian	○ Never	○ Once	○ 2-3 times	○ 4+ times
**PF3**	Have you seen your mother/female guardian being beaten by any of the family members?	○ Never	○ Once	○ 2-3 times	○ 4+ times

Have you ever had a boyfriend? ○ No ○ Yes ○ Don't Know

Physical/Emotional IPV (pIPV/eIPV)
Please make an X in the bubble of the best answer.

**pIPV1**	How many times within the past 12 months has your current or a previous boyfriend slapped you or thrown something at you which could hurt you?	○ Never	○ Once	○ 2-3 times	○ 4+ times

**pIPV2**	How many times within the past 12 months has your current or a previous boyfriend pushed or shoved you?	○ Never ○ Once ○ 2-3 times ○ 4+ times
**pIPV3**	How many times within the past 12 months has your current or a previous boyfriend hit you with a fist or with something else which could hurt you?	○ Never ○ Once ○ 2-3 times ○ 4+ times
**pIPV4**	How many times within the past 12 months has your current or a previous boyfriend kicked, dragged, beat, choked, or burned you?	○ Never ○ Once ○ 2-3 times ○ 4+ times
**pIPV5**	How many times within the past 12 months has your current or a previous boyfriend threatened to use or actually used a gun, knife or other weapon (any object that can be used to inflict harm) against you?	○ Never ○ Once ○ 2-3 times ○ 4+ times
**eIPV1**	How many times within the past 12 months has your current or a previous boyfriend insulted you or made you feel bad about yourself?	○ Never ○ Once ○ 2-3 times ○ 4+ times
**eIPV2**	How many times within the past 12 months has your current or a previous boyfriend made fun of or humiliated you in front of other people?	○ Never ○ Once ○ 2-3 times ○ 4+ times
**eIPV3**	How many times in the past 12 months has your current or a previous boyfriend threatened to hurt you or done things to scare or intimidate you on purpose, for example by the way he looked at you, by yelling and smashing things?	○ Never ○ Once ○ 2-3 times ○ 4+ times

**Sexual Behavior and First Time Assault/Coercion and Alcohol Questions**		
**F1**	Have you ever had a sexual intercourse?	○ Yes ○ No ○ Don't Know ○ Refused
**F2**	How old were you when you had sex for the first time?	[ ][ ] ○ Don't Know ○ Refused ○ N/A
**F3**	Has anyone ever given you money, food, gifts, or any favors to have sex with them?	○ Yes ○ No ○ Don't Know ○ Refused
**F4**	How many times do you have a drink containing alcohol?<br><br>Alcohol includes things like beer, wine, chang'aa, palm wine, whiskey, gin, vodka, etc.	○ Never<br>○ Once<br>○ Monthly or less<br>○ A few times per month<br>○ A few times per week<br>○ Daily<br>○ Don't Know<br>○ Refused

**F5**	In the past 30 days, on how many days did you drink alcohol to the point that you become drunk	☐☐ ○ N/A ○ Refused
**F6**	How many times do you use drugs, such as marijuana, glue sniffing, heroin, cocaine?	○ Never<br>○ Once<br>○ Monthly or less<br>○ A few times per month<br>○ A few times per week<br>○ Daily<br>○ N/A<br>○ Refused

\

**Questions Regarding Boyfriend**	
**BF1**	In the past 12 months, how many times has a current or a previous boyfriend ever physically forced you to have sex when you did not want to?<br>○ Never ○ Once ○ 2-3 times ○ 4+ times ○ N/A
**BF2**	In the past 12 months, how many times has your current or a previous boyfriend used threats or intimidation to get you to have sex when you did not want to?<br>○ Never ○ Once ○ 2-3 times ○ 4+ times ○ N/A
**BF3**	In the past 12 months, how many times has a current or your previous boyfriend ever forced you to do something else sexual that you did not want to do?<br>○ Never ○ Once ○ 2-3 times ○ 4+ times ○ N/A

**Non-Partner Sexual Violence Questions**		
**NPSV1**	In the past 12 months, how many times has a man who is NOT your boyfriend forced or persuaded you to have sex against your will?	○ Never ○ Once ○ 2-3 times ○ 4+ times

**NPSV1**	Who forced or persuaded you to have sex against your will?<br><br>Please make an X in the bubbles of all answers that apply.	○ Did not force/persuade others to have sex against their will<br>○ Family member/relative<br>○ Authority figure such as teacher or police<br>○ Other<br>○ Did not know them<br>○ Refused<br>○ N/A<br><br>Please Specify Other<br>[ ] [ ] [ ] [ ] [ ] [ ] [ ] [ ] [ ] [ ] [ ] [ ] [ ] [ ] [ ] [ ] [ ] [ ] [ ] [ ]
**NPSV2**	In the past 12 months, how many times has any man or boy who is NOT your boyfriend tried to force you to have sex against your will and did not succeed?	○ Never ○ Once ○ 2-3 times ○ 4+ times
**NPSV3**	In the past 12 months how many times has any man or boy who is NOT your boyfriend forced you to have sex against your will when you were too drunk or drugged to refuse?	○ Never ○ Once ○ 2-3 times ○ 4+ times
**NPSV4**	In the last 12 months how many times did two or more men or boys force you to have sex with them at the same time against your will?	○ Never ○ Once ○ 2-3 times ○ 4+ times
**NPSV5**	In the past 12 months how many times did two or more men or boys force you to have sex with them at the same time against your will when you were too drunk or drugged to refuse?	○ Never ○ Once ○ 2-3 times ○ 4+ times
**NPSV6**	In the past 12 months how many times was there an occasion when you agreed to have sex with one man or boy and one or more others who you had not agreed to have sex with forced you to have sex with them as well?	○ Never ○ Once ○ 2-3 times ○ 4+ times

**NPSV7**	Where were you the most recent time someone forced you to have sex?	◯ My home<br>◯ Perpetrator's home<br>◯ Someone else's home<br>◯ Party<br>◯ Public Event<br>◯ School<br>◯ Car/Bus<br>◯ Travelling by foot<br>◯ Other locations<br>◯ Don't Know<br>◯ Refused<br>Please Specify Other<br>[ ]
**NPSV8**	What was going on just before this happened?	◯ On date<br>◯ At party<br>◯ Fighting with perpetrator<br>◯ Fighting with family<br>◯ Going to public event<br>◯ At public event<br>◯ Going for water<br>◯ Going shopping<br>◯ Other situations<br>◯ Don't Know<br>◯ N/A<br>Please Specify Other<br>[ ]
**NPSV9**	About what time of day did this happen?	◯ Morning<br>◯ Afternoon<br>◯ Evening<br>◯ Late at night<br>◯ Don't Know<br>◯ N/A

**NPSV10**	Have you ever gotten pregnant as a result of being forced to have sex?	○ Yes ○ No ○ Refused to answer ○ N/A
**NPSV11**	Have you ever gone for an HIV test as a result of being forced to have sex?	○ Yes ○ No ○ Refused to answer ○ N/A
**NPSV12**	How many times in your life have you been forced to have sex against your will?	○ Never ○ Once ○ Don't Know ○ Refused to answer<br>Write number if 2 times or more: ☐☐ ○ N/A
**NPSV13**	If you know any of the people who did this to you, can you tell me the number of times they forced you to have sex?<br><br>Please make an X in the bubbles of all answers that apply	○ Boyfriend or Partner ☐☐<br>○ Family member or relative ☐☐<br>○ Authority Figure (teacher/police) ☐☐<br>○ I did not know them ☐☐<br>○ Refused to answer<br>○ N/A
**NPSV14**	Did you ever tell anybody about any of these incidents?	○ Yes ○ No ○ Don't Know ○ N/A

**NPSV15**	Who did you tell?<br><br>Please mark all that apply	○ No One<br>○ Friend<br>○ Father<br>○ Mother<br>○ Brother<br>○ Sister<br>○ Boyfriend/Romantic Partner<br>○ Teacher<br>○ Police<br>○ Other Male Relative<br>○ Other Female Relative<br>○ Other<br>○ None<br>○ Refused
	Other Male Relative:	
	Other Female Relative:	
	Other Person Told:	
**NPSV16**	Were there any sexual incidents that you did not tell anyone about?	○ Yes ○ No ○ None ○ Refused
**NPSV17**	Did you try to seek professional help for any of these incidents, like from a clinic or other organization?	○ Yes ○ No ○ None ○ Refused
**NPSV18**	Were you successful in receiving any professional help for any of these incidents, like from a clinic or other organization?	○ Yes ○ No ○ None ○ Refused

Self-Efficacy: Self-Efficacy Questionnaire for Children (SEQ-C)
Please make an X in the bubble of the best answer

**1.**	How well can you get teacher to help you when you get stuck on schoolwork?	○ 1 Not at all	○ 2 A little bit	○ 3 About average	○ 4 Well	○ 5 Very Well
**2.**	How well can you express your opinion when other classmates disagree with you?	○ 1 Not at all	○ 2 A little bit	○ 3 About average	○ 4 Well	○ 5 Very Well
**3.**	How well do you succeed in cheering yourself up when an unpleasant event has happened?	○ 1 Not at all	○ 2 A little bit	○ 3 About average	○ 4 Well	○ 5 Very Well

**4.**	How well can you study when there are other interesting things to do?	○ 1 Not at all	○ 2 A little bit	○ 3 About average	○ 4 Well	○ 5 Very Well
**5.**	How well do you succeed in becoming calm again when you are very scared?	○ 1 Not at all	○ 2 A little bit	○ 3 About average	○ 4 Well	○ 5 Very Well
**6.**	How well can you become friends with other children?	○ 1 Not at all	○ 2 A little bit	○ 3 About average	○ 4 Well	○ 5 Very Well
**7.**	How well can you study a chapter for a test?	○ 1 Not at all	○ 2 A little bit	○ 3 About average	○ 4 Well	○ 5 Very Well
**8.**	How well can you have a chat with an unfamiliar person?	○ 1 Not at all	○ 2 A little bit	○ 3 About average	○ 4 Well	○ 5 Very Well
**9.**	How well can you prevent to become nervous?	○ 1 Not at all	○ 2 A little bit	○ 3 About average	○ 4 Well	○ 5 Very Well
**10.**	How well do you succeed in finishing all your homework every day?	○ 1 Not at all	○ 2 A little bit	○ 3 About average	○ 4 Well	○ 5 Very Well
**11.**	How well can you work in harmony with your classmates?	○ 1 Not at all	○ 2 A little bit	○ 3 About average	○ 4 Well	○ 5 Very Well
**12.**	How well can you control your feelings?	○ 1 Not at all	○ 2 A little bit	○ 3 About average	○ 4 Well	○ 5 Very Well
**13.**	How well can you pay attention during every class?	○ 1 Not at all	○ 2 A little bit	○ 3 About average	○ 4 Well	○ 5 Very Well
**14.**	How well can you tell other children that they're doing something you don't like?	○ 1 Not at all	○ 2 A little bit	○ 3 About average	○ 4 Well	○ 5 Very Well
**15.**	How well can you give yourself a pep-talk when you feel low?	○ 1 Not at all	○ 2 A little bit	○ 3 About average	○ 4 Well	○ 5 Very Well
**16.**	How well do you succeed in understanding all subjects in school?	○ 1 Not at all	○ 2 A little bit	○ 3 About average	○ 4 Well	○ 5 Very Well
**17.**	How well can you tell a funny event to a group of children?	○ 1 Not at all	○ 2 A little bit	○ 3 About average	○ 4 Well	○ 5 Very Well
**18.**	How well can you tell a friend that you don't feel well?	○ 1 Not at all	○ 2 A little bit	○ 3 About average	○ 4 Well	○ 5 Very Well

**19.**	How well do you succeed in satisfying your parents/guardians with your schoolwork?	○ 1 Not at all	○ 2 A little bit	○ 3 About average	○ 4 Well	○ 5 Very Well
**20.**	How well do you succeed in staying friends with other children?	○ 1 Not at all	○ 2 A little bit	○ 3 About average	○ 4 Well	○ 5 Very Well
**21.**	How well do you succeed in suppressing unpleasant thoughts?	○ 1 Not at all	○ 2 A little bit	○ 3 About average	○ 4 Well	○ 5 Very Well
**22.**	How well do you succeed in passing a test?	○ 1 Not at all	○ 2 A little bit	○ 3 About average	○ 4 Well	○ 5 Very Well
**23.**	How well do you succeed in preventing quarrels with other children?	○ 1 Not at all	○ 2 A little bit	○ 3 About average	○ 4 Well	○ 5 Very Well
**24.**	How well do you succeed in not worrying about things that might happen?	○ 1 Not at all	○ 2 A little bit	○ 3 About average	○ 4 Well	○ 5 Very Well

Self-Esteem: Rosenberg's Self Esteem Scale
Please make an X in the bubble of the best answer

**1.**	I feel that I am a person of worth, at least on an equal plane with others	○ Strongly Agree	○ Agree	○ Disagree	○ Strongly Disagree
**2.**	I feel that I have a number of good qualities	○ Strongly Agree	○ Agree	○ Disagree	○ Strongly Disagree
**3.**	All in all, I am inclined to feel that I am a failure	○ Strongly Agree	○ Agree	○ Disagree	○ Strongly Disagree
**4.**	I am able to do things as well as most other people	○ Strongly Agree	○ Agree	○ Disagree	○ Strongly Disagree
**5.**	I feel I do not have much to be proud of	○ Strongly Agree	○ Agree	○ Disagree	○ Strongly Disagree
**6.**	I take a positive attitude toward myself	○ Strongly Agree	○ Agree	○ Disagree	○ Strongly Disagree
**7.**	On the whole, I am satisfied with myself	○ Strongly Agree	○ Agree	○ Disagree	○ Strongly Disagree
**8.**	I wish I could have more respect for myself	○ Strongly Agree	○ Agree	○ Disagree	○ Strongly Disagree

**9.**	I certainly feel useless at times	◯ Strongly Agree	◯ Agree	◯ Disagree	◯ Strongly Disagree
**10.**	At times, I think I am no good at all	◯ Strongly Agree	◯ Agree	◯ Disagree	◯ Strongly Disagree

Ideas About Gender Relations

Now I would like to ask your opinion on some statements on relations between men and women, can you tell me if you strongly agree, agree, disagree or strongly disagree with the following statements:

**1.**	A woman should listen to her husband	◯ Strongly Agree	◯ Agree	◯ Disagree	◯ Strongly Disagree
**2.**	A woman has to teach her man to respect her	◯ Strongly Agree	◯ Agree	◯ Disagree	◯ Strongly Disagree
**3.**	A woman should choose her own friends even if her boyfriend or husband disagrees	◯ Strongly Agree	◯ Agree	◯ Disagree	◯ Strongly Disagree
**4.**	Men should share the work around the home such as doing the dishes or cleaning or cooking	◯ Strongly Agree	◯ Agree	◯ Disagree	◯ Strongly Disagree
**5.**	Sometimes a man may have a good reason to hit his girlfriend	◯ Strongly Agree	◯ Agree	◯ Disagree	◯ Strongly Disagree
**6.**	A woman can refuse to have sex with her husband if she does not want it for any reason	◯ Strongly Agree	◯ Agree	◯ Disagree	◯ Strongly Disagree
**7.**	If a wife does something wrong she should expect her husband to punish her	◯ Strongly Agree	◯ Agree	◯ Disagree	◯ Strongly Disagree
**8.**	A woman has to know how to look after herself as she cannot rely on her man to care for her	◯ Strongly Agree	◯ Agree	◯ Disagree	◯ Strongly Disagree
**9.**	A man cannot control himself when he wants sex	◯ Strongly Agree	◯ Agree	◯ Disagree	◯ Strongly Disagree
**10.**	A woman should expect to be taught how to behave by her boyfriend	◯ Strongly Agree	◯ Agree	◯ Disagree	◯ Strongly Disagree
**11.**	A woman should not expect the fathers of her children to give her money	◯ Strongly Agree	◯ Agree	◯ Disagree	◯ Strongly Disagree
**12.**	If a woman drinks alcohol and wears miniskirts she is asking for trouble	◯ Strongly Agree	◯ Agree	◯ Disagree	◯ Strongly Disagree
